# High Intensity Physical Exercise and Pain in the Neck and Upper Limb among Slaughterhouse Workers: Cross-Sectional Study

**DOI:** 10.1155/2014/218546

**Published:** 2014-01-09

**Authors:** Emil Sundstrup, Markus D. Jakobsen, Kenneth Jay, Mikkel Brandt, Lars L. Andersen

**Affiliations:** ^1^National Research Centre for the Working Environment, 2100 Copenhagen, Denmark; ^2^Institute for Sports Science and Clinical Biomechanics, University of Southern Denmark, 5230 Odense, Denmark

## Abstract

Slaughterhouse work involves a high degree of repetitive and forceful upper limb movements and thus implies an elevated risk of work-related musculoskeletal disorders. High intensity strength training effectively rehabilitates musculoskeletal disorders among sedentary employees, but less is known about the effect among workers with repetitive and forceful work demands. Before performing randomized controlled trials it may be beneficial to assess the cross-sectional connection between exercise and musculoskeletal pain. We investigated the association between high intensity physical exercise and pain among 595 slaughterhouse workers in Denmark, Europe. Using logistic regression analyses, odds ratios for pain and work disability as a function of physical exercise, gender, age, BMI, smoking, and job position were estimated. The prevalence of pain in the neck, shoulder, elbow, and hand/wrist was 48%, 60%, 40%, and 52%, respectively. The odds for experiencing neck pain were significantly lower among slaughterhouse workers performing physical exercise (OR = 0.70, CI: 0.49–0.997), whereas the odds for pain in the shoulders, elbow, or hand/wrist were not associated with exercise. The present study can be used as general reference of pain in the neck and upper extremity among slaughterhouse workers. Future studies should investigate the effect of high intensity physical exercise on neck and upper limb pain in slaughterhouse workers.

## 1. Introduction

Musculoskeletal disorders constitute the most common occupational disease in the European union and in the United States [1, 2]. The consequences can be pervasive to both employees and society, affecting individual health and wellbeing, and impose a substantial socioeconomic burden due to increased sickness absence, disability pension, and loss of productivity [3–5]. Physical exposures such as repetitive and forceful muscle work, lack of sufficient recovery, precision demands, and awkward postures are risk factors for neck and upper limb musculoskeletal pain [5–7].

Slaughtering and meat processing operations involve a high degree of repetitive and forceful upper limb movements and thus implies an elevated risk of work-related musculoskeletal disorders [8, 9]. In line with this, the rate of nonfatal occupational injuries and illnesses for workers engaged in animal slaughtering is more than twice as high as the US national average, and the number of cases with days away from work, job transfer, or restriction is almost three times the national average [2]. To effectively prevent or rehabilitate musculoskeletal pain among this occupational group, the prevalence and associates of pain in the neck, shoulder, and arm must be identified.

A strategy to reduce or prevent work-related musculoskeletal pain may be to increase the workers physical capacity through physical exercise. Previous studies from our research group have shown promising and effective reductions in neck/shoulder/arm pain in response to 10–20 weeks of kettlebell training [10, 11], strength training using elastic rubber bands [12, 13] or free weights [14–16] in office workers and laboratory technicians. However, office workers and laboratory technicians have less strenuous working conditions than slaughterhouse workers, and our previous findings may therefore not apply to other occupational groups. No previous studies have investigated the effect of physical exercise among slaughterhouse workers, perhaps because the time pressure in this occupation makes physical exercise at the workplace difficult to implement. Thus, before performing randomized controlled trials among slaughterhouse workers it may be beneficial to assess the cross-sectional association between leisure-time physical exercise and pain.

The aim of this study was to investigate in a cross-sectional questionnaire survey the association between high intensity physical exercise and musculoskeletal pain in the neck and upper extremity among slaughterhouse workers. We hypothesized that slaughterhouse workers engaged in high intensity physical exercise would have better odds of being pain-free than their inactive colleagues.

## 2. Methods

### 2.1. Study Design

A cross-sectional questionnaire survey concerning musculoskeletal pain and physical exercise was conducted among 595 slaughterhouse workers in Denmark, Europe. The Danish Data Protection Agency was notified of and registered the study (registration number 2010-54-1106). According to Danish law, questionnaire- and register-based studies need neither approval from ethical and scientific committees nor informed consent [17, 18]. However, prior to data collection, an informational meeting was held at the two participating slaughterhouses.

### 2.2. Questionnaire and Outcomes

The questionnaire was sent out to 645 slaughterhouse workers of which 595 (92%) responded. The questionnaire consisted of 25 items concerning demographics, regional pain intensity, physical exercise habits, work-related characteristics, and general health.

Regional pain intensity during the last three months was rated subjectively using the 0–10 modified VAS scale, where 0 indicates “no pain at all” and 10 indicates “worst pain imaginable” [12, 19]. The neck, shoulder, elbow, and hand/wrist regions were defined by drawings from the Nordic Questionnaire of Musculoskeletal Pain [20]. Subsequently pain rated on the numerical rating scale was dichotomized into two groupings; “pain” as a score of 3–10 and “no or little pain” as 0–2 [21].

The following question defined high intensity physical exercise: “*Do you perform one of the following types of strenuous physical exercises at least once a week,*” with yes/no response option to each of the following: (1) strength training, (2) aerobic fitness, (3) ball games, or (4) any other type of strenuous physical exercise. Subsequently physical exercise was dichotomized into two groupings: “physically active” identified by performing a weekly minimum of one of the 4 options and “physically inactive” as not performing any weekly physical exercise.

Participants were asked about year of birth, height and weight, seniority, and primary job function. Primary job function was further dichotomized into “meat cutters” and “meat packers.” Smoking habits were identified by the question “do you smoke?”—“yes” or “no.” Further, work disability was present if the participant stated at least “*some*” work disability scoring on a five-point scale: “*not at all,*” *“a little,” “some,” “much,*” and “*very much,*” when asked the question “*during the last 3 months, did you have any difficulty performing your work due to pain in the shoulder, arm, or hand.*”

## 3. Statistical Analysis

Using logistic regression analyses (PROC GENMOD of SAS version 9.2), we estimated the odds for pain in the four different body regions and work disability. We included physical exercise, gender, age, BMI, smoking, and job position as independent variables in the model. Odds ratios (OR) and 95% confidence intervals (95% CI) were calculated for the four body regions and work disability. Further, Spearman's correlation coefficient was calculated to determine the association between pains in the different body regions. *P* values less than 0.05 were deemed statistically significant.

## 4. Results

### 4.1. Regional Pain Intensity

Anthropometric data, pain, and disability are illustrated in Table 1. The prevalence of pain in the neck, shoulder, elbow, and hand/wrist was 48%, 60%, 40%, and 52%, respectively (Table 1). Additionally, pain in one region was associated with pain in the three other regions, evidenced by moderate Spearman's correlation coefficients (*r*_s_ 0.50–0.69, *P* < 0.0001) (Table 2).

### 4.2. Odds Ratios

Table 3 summarizes the OR estimates for experiencing pain in the four different body regions or work disability as a function of physical exercise, gender, age, BMI, smoking, and job position.

The odds for experiencing neck pain were lower among slaughterhouse workers performing physical exercise (OR = 0.7, CI: 0.49–0.997; *P* = 0.048). However, the odds for experiencing pain in the shoulders, elbow, or hand/wrist were not significantly lower among workers performing physical exercise (*P* = 0.35–0.89).

Females demonstrated higher odds for hand/wrist pain (OR = 2.27; CI: 1.19–4.35) than males, and smokers had higher odds for neck, elbow, and hand/Wrist pain and disability (OR ranging from 1.45 to 1.76).

Job function was associated with regional pain and disability. Meatpackers demonstrated more increased odds for suffering from pain in the neck (OR = 2.1; CI: 1.35–3.25) and shoulders (OR = 2.19; CI: 1.38–3.47) and disability (OR = 1.95, CI: 1.27–2.99) than meat cutters.

Additionally, neither age nor BMI significantly affected the odds for experiencing pain or disability among the slaughterhouse workers.

## 5. Discussion

Our cross-sectional study shows that physical exercise is associated with reduced risk of experiencing neck pain in slaughterhouse workers. However, pain in the shoulders, elbow, and hand/wrist was not significantly associated with regular physical exercise.

The prevalence of pain in the neck, shoulders, elbow and hand/wrist among workers engaged in slaughtering and meat processing operations was high, affecting between 40% and 60%. We hypothesized that the risk for experiencing pain in the neck and upper limb would be diminished among workers engaged in physical exercise compared to inactive colleagues. However, physical exercise was only associated with lower odds for experiencing pain in the neck, whereas no association with pain in the shoulder, elbow, and hand/wrist was found. Due to the cross-sectional nature of this study we cannot infer anything about causality between physical exercise and pain; however, promising and effective reductions in neck/shoulder/arm pain have been demonstrated in response to physical exercise among sedentary job groups [10, 12]. In particular, strength training targeting the painful regions has shown clinical relevant reductions in neck/shoulder pain and seems superior to aerobic fitness in pain management among office workers [22]. General physical exercise has also shown beneficial effects among office workers [21]. However, office workers and laboratory technicians have less strenuous working conditions than slaughterhouse workers, and our previous findings may therefore not be directly transferable to this occupational group. Further, repetitive and forceful muscle work may—in theory—hinder adequate recovery between resistance training sessions and workdays. The cross-sectional association between physical exercise and neck pain indicates that physical exercise may lead to reductions in neck pain among slaughterhouse workers. However, randomized controlled trials among slaughterhouse workers investigating the effect of high intensity physical exercise, for example, strength training targeting the neck, shoulders, elbow, and hand/wrist, are needed.

### 5.1. Additional Findings

Pain in one region was associated with pain in the other regions, stressing the highly repetitive and high force exposure of these body regions during work as risk factors for developing musculoskeletal disorders in several regions of the upper extremity. In addition, pain in one area may lead to altered or compensating moving patterns of the arm possibly affecting mechanical loading in the adjacent regions of the neck, shoulder, and arm. In line with this, Andersen and coworkers [23] found that chronic pain in the neck/shoulders among healthcare workers increased the risk for developing chronic pain in other body regions, possibly due to a combination of central sensitization and compensatory lifting patterns.

Meat packing tasks were associated with a higher risk for experiencing neck and shoulder pain and disability than meat cutting operations. Thus it seems that repetitive lifting tasks, during meat packing, primarily expose the neck and shoulder whereas meat cutting operations involve a great portion of repetitive movements of the hand/wrist and elbow. Whether meat packing is a stronger risk factor than meat cutting and how other contributing factors influence pain and job position are beyond the scope of this study. It should however be mentioned that injured meat cutters are often transferred to job tasks involving meat packing, as this job task is considered less exposed than meat cutting. Thus, a natural selection towards specific work tasks may occur.

### 5.2. Limitations and Strength

This is the first study to investigate associations between physical exercise and pain intensity in slaughterhouse workers. The cross-sectional study design is a limitation as we cannot infer anything about causality. However, the study should be seen as a predecessor for future randomized controlled trials investigating effective pain management among workers engaged in repetitive and physical jobs. Because this study is based on questionnaire replies the risk of common rater bias exists; that is, the individual may reply negatively or positively to questions in general. On the other hand, pain is a subjective experience. A strength is that we used the validated Nordic Questionnaire of Musculoskeletal Pain with pictures defining the different body regions of interest [20]. The study population limits the generalizability of our findings to slaughterhouse workers. However, different job groups may respond differently to physical exercise, and performing such analyses among specific job groups is therefore of paramount importance.

### 5.3. Conclusion. 

Physical exercise was associated with lower odds for experiencing pain in the neck, whereas no significant association between exercise and pain in the shoulder, elbow, and hand/wrist was found. Future studies should in randomized controlled trials investigate the effect of high intensity physical exercise on neck and upper limb pain in workers exposed to highly repetitive and forceful work.

## Figures and Tables

**Table 1:** Characteristics of the participants.

Age (years)	44 (9.8)
BMI (kg·m^−2^)	27 (4.6)
Male/female	89%/11%
Meat cutter/packer	74%/26%
Physical exercise	49%
Neck pain	48%
Shoulder pain	60%
Elbow pain	40%
Hand/wrist pain	52%
Work disability	38%
Smoking	37%

**Table 2:** The association between pain in neck, shoulders, elbow, and hand/wrist is estimated by Spearman's correlation coefficients (*r*_*s*_). All *r*_*s*_ are between 0.5 and 0.7 and thus considered as moderate correlations.

	Neck pain	Shoulder pain	Elbow pain	Hand/wrist pain
Neck pain	1			
Shoulder pain	0.69 (*P* < 0.0001)	1		
Elbow pain	0.52 (*P* < 0.0001)	0.51 (*P* < 0.0001)	1	
Hand/wrist pain	0.50 (*P* < 0.0001)	0.55 (*P* < 0.0001)	0.52 (*P* < 0.0001)	1

**Table 3:** Odds ratios (OR) for pain in the four body regions and disability are calculated with gender, physical exercise, age, BMI, smoking, and job function as independent variables. Statistical significant odds ratios (*P* < 0.05) are marked with bold, OR (95% CI).

	Neck	Shoulders	Elbow	Hand/wrist	Work disability
Age	1.01 (0.99–1.03)	1.01 (0.99–1.03)	1.03 (1.01–1.05)	1.00 (0.98–1.02)	1.00 (0.98–1.02)
BMI	1.00 (0.97–1.04)	1.01 (0.98–1.06)	0.99 (0.95–1.03)	0.98 (0.94–1.02)	1.03 (0.99–1.07)
Gender					
Male	1	1	1	1	1
Female	1.62 (0.86–3.04)	1.77 (0.89–3.52)	1.24 (0.67–2.29)	**2.27 (1.19–4.35)**	1.83 (0.99–3.38)
Physical exercise					
No	1	1	1	1	1
Yes	**0.70 (0.49–1.00)**	1.04 (0.72–1.49)	0.98 (0.68–1.40)	0.84 (0.59–1.2)	0.89 (0.62–1.28)
Smoking					
No	1	1	1	1	1
Yes	**1.76 (1.21–2.55)**	1.39 (0.95–2.03)	**1.54 (1.06–2.23)**	**1.53 (1.06–2.2)**	**1.45 (1.00–2.11)**
Job position					
Meat cutter	1	1	1	1	1
Meat packer	**2.10 (1.35–3.25)**	**2.19 (1.38–3.47)**	1.51 (0.98–2.32)	1.21 (0.79–1.86)	**1.95 (1.27–2.99)**
